# The genomic signal of local environmental adaptation in *Aedes aegypti* mosquitoes

**DOI:** 10.1111/eva.13199

**Published:** 2021-02-26

**Authors:** Kelly L. Bennett, W. Owen McMillan, Jose R. Loaiza

**Affiliations:** ^1^ Smithsonian Tropical Research Institute Balboa Ancon Republic of Panama; ^2^ Instituto de Investigaciones Científicas y Servicios de Alta Tecnología Panamá Republic of Panama; ^3^ Programa Centroamericano de Maestría en Entomología Universidad de Panamá Panamá Republic of Panama

**Keywords:** *Aedes* mosquitoes, arboviral disease landscape, environmental association analysis, hybridization capture‐based target enrichment, local adaptation, Panama

## Abstract

Local adaptation is important when predicting arthropod‐borne disease risk because of its impacts on vector population fitness and persistence. However, the extent that vector populations are adapted to the environment generally remains unknown. Despite low population structure and high gene flow in *Aedes aegypti* mosquitoes across Panama, excepting the province of Bocas del Toro, we identified 128 candidate SNPs, clustered within 17 genes, which show a strong genomic signal of local environmental adaptation. This putatively adaptive variation occurred across fine geographical scales with the composition and frequency of candidate adaptive loci differing between populations in wet tropical environments along the Caribbean coast and dry tropical conditions typical of the Pacific coast. Temperature and vegetation were important predictors of adaptive genomic variation in *Ae. aegypti* with several potential areas of local adaptation identified. Our study lays the foundations of future work to understand whether environmental adaptation in *Ae. aegypti* impacts the arboviral disease landscape and whether this could either aid or hinder efforts of population control.

## 1 INTRODUCTION

The establishment and persistence of vectors within new geographical locations poses a serious threat from emerging and endemic arboviral diseases (Kilpatrick & Randolph, 2012; Weaver & Reisen, 2010). For example, shifts in the distribution of ticks and *Culex* mosquitoes are linked to the rise of West Nile Virus and tick‐borne encephalitis viruses within North America (Artsob et al., 2009; Gasmi et al., 2018; Sonenshine, 2018). In addition, the introduction of invasive *Aedes* mosquitoes has facilitated the recent spread of Zika and Chikungunya viruses throughout the Americas (Musso et al., 2018; Weaver, 2014). Although introduced vector populations are unlikely to be at their fitness optimum when first confronted with a new environment, local adaptation may play a large role in disease dynamics as vectors adapt to their environment, increase their relative fitness and acquire new traits, thus potentially increasing the threat of human arboviruses. There are few examples where local environmental adaptation has been investigated in *Aedes* mosquitoes, which have involved large‐scale comparisons between source and invasive populations (Sherpa, Guéguen, et al., 2019), northern and southern latitudes (Sherpa, Blum, et al., 2019) and across the Caribbean islands (Sherpa et al., 2018). However, *Aedes* local adaptation to the environment has not been discussed across a fine geographical scale.

The importance of adaptation for human disease is exemplified in *Aedes aegypti*'s evolution to human commensalism and the establishment of a number of arboviruses worldwide (Powell & Tabachnick, 2013). This mosquito has undergone behavioural and genetic changes in comparison to its ancestral African form, including the evolution of house‐entering behaviour and a preference for human odour and blood‐feeding (Brown et al., 2014; McBride et al., 2014; Trpis & Hausermann, 1978). The adaptation of *Ae. aegypti* to exploit human environments has allowed for the spread of zoonotic arboviral diseases from forest animals to humans and promoted invasiveness through human‐assisted dispersal (Powell & Tabachnick, 2013). Although it is known that the environment is important in driving and altering the life‐history traits of *Aedes* mosquitoes (Brady et al., 2013; Leisnham & Juliano, 2010; Tun‐Lin et al., 2000), there remains a lack of understanding on how their genomic background changes across a heterogeneous environment. A landscape genomics approach forms a crucial first step to identify population structure associated with the environment and to narrow down candidate genomic targets for further investigation of local environmental adaptation.

Here we characterize genome‐wide variation in *Ae. aegypti* across Panama and use these data to explore the potential for local adaptation associated with environmental heterogeneity. Panama provides an ideal opportunity to understand how a combination of gene flow and differential adaptation to local climate conditions interacts to affect the disease landscape. Panama is a small Neotropical country, measuring just 772 km east to west and 185 km north to south, but provides a wealth of contrasting climatic conditions and discrete environments. This is largely owing to its geography as a narrow isthmus flanked by the Caribbean Sea and Pacific Ocean as well as the Cordillera Central mountain range, which acts as a north–south divide. Panama is also a hub of international shipping trade, providing an important route of *Aedes* mosquito invasion into the Americas. Panama's worldwide connections have potentially facilitated multiple introductions of the invasive *Ae. aegypti* mosquito dating back to the 18th century in association with the global shipping trade (Bennett et al., 2016; Eskildsen et al., 2018; Powell & Tabachnick, 2013). In addition, the Pan‐American highway bisects the country, stretches almost 48,000 km throughout mainland America and provides an important conduit for the human‐assisted dispersal of *Aedes* mosquitoes (Bennett et al., 2019; Miller & Loaiza, 2015).

We first investigate how genomic variation in *Ae. aegypti* is distributed across Panama. We then examine the possibility that *Ae. aegypti* are adapted to heterogeneous environments by identifying loci with a genomic signal of local adaptation that are associated with discrete environmental conditions. Whether the environment informs population structure is an important consideration for vector control that will also provide insight into the global spatial heterogeneity of viral transmission.

## 2 MATERIALS AND METHODS

### 2.1 Mosquito sampling

*Aedes* mosquitoes were collected with oviposition traps placed across 14 settlements and six provinces of Panama across the rainy season months of May to November in 2017 (Table S1). Within each settlement, twenty oviposition traps were placed at least 300 metres apart to promote sampling independence. Values of pairwise identity by descent calculated between individuals from the same settlement within PLINK v1.90 (Purcell et al., 2007) were all below the 0.5 expected of full siblings. Oviposition traps at each location were removed and checked for mosquito larvae after five days. Immature stages of *Aedes* from each trap were reared to adulthood as separate collections in the laboratory, identified using the morphological key of Rueda (2004) and stored in absolute ethanol at −20°C.

### 2.2 Genomics data

**FIGURE 1:**
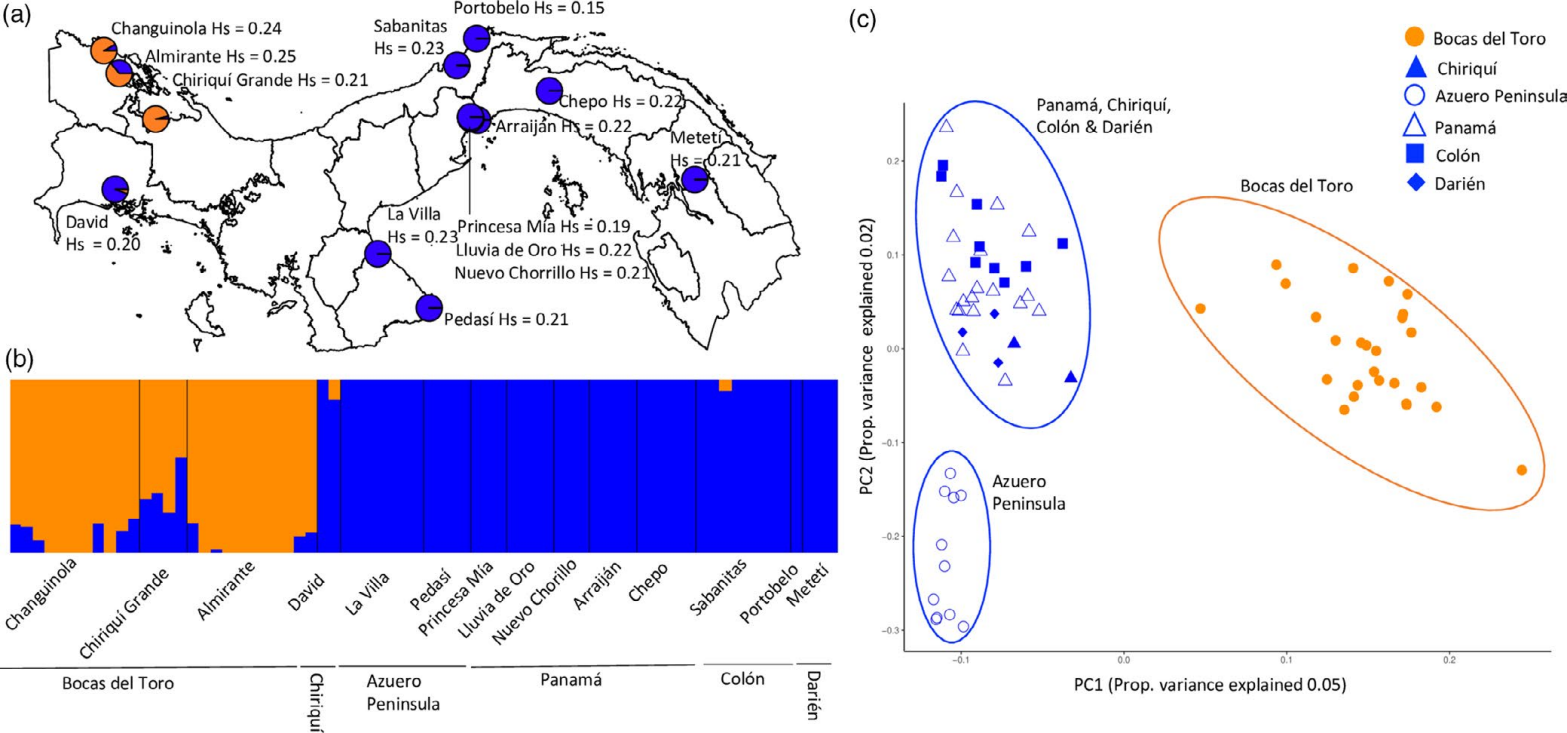
Marked population structure across the Isthmus of Panama: (a) Admixture proportions of *K* = 2 populations in relation to sampling locations and population heterozygosity Hs of *Aedes aegypti* across Panama as determined by FastStructure for 371,307 SNPs. FastStructure assigns each individual to one or more *K* populations, as indicated by its colour. Genetically similar populations share the same colour or similar admixture composition on comparison. (b) The FastStructure plot comparing the genomic composition of individuals of *Ae. aegypti* across the different regions of Panama for *K* = 2 populations. (c) PCA of all 371,307 *Ae. aegypti* SNPs grouped by region

DNA was extracted from 70 *Ae. aegypti* (Figure 1a), representing populations subject to different environmental conditions using a modified phenol chloroform method (Surendran et al., 2013). Only one individual was processed from each oviposition trap to avoid sampling siblings. To identify putative regions involved in the local adaption of *Ae. aegypti*, 26.74 Mb of the AaeL3 exome was targeted for capture. For each sample, 100 ng DNA was mechanically sheared to fragment sizes of ~350–500 base pairs and processed to add Illumina adapters using the Kapa HyperPrep Kit. Amplified libraries were assessed on a Bioanalyser and Qubit before 24 uniquely barcoded individuals each were pooled to a combined mass of 1 μg to create three libraries of 24 individuals for hybridization. Sequence capture of exonic regions was performed on each pool according to the NimbleGen SeqCap EZ HyperCap workflow and using custom probes designed by Roche for the regions we specified (Dataset S1).

Low‐quality base calls (<20) and Illumina adapters were trimmed from sequence ends with TrimGalore (Krueger, 2015), before alignment to the *Ae. aegypti* AaeL5 reference genome with Burrows–Wheeler aligner (Li & Durbin, 2010). Read duplicates were removed with BamUtil. Sequence reads were processed according to the GATK best practice recommendations, trained with a hard‐filtered subset of SNPs using online recommendations (https://gatkforums.broadinstitute.org/gatk/discussion/2806/howto‐apply‐hard‐filters‐to‐a‐call‐set). SNPs were called with a heterozygosity prior 0.0014, a previously reported value of theta (Rašić et al., 2014). Filters applied to the resulting SNP data set using VCFtools (Danecek et al., 2011) included a minimum quality of 30, minimum depth of 30, minimum mean depth of 20, maximum 5% missing data across individuals and a minor allele frequency ≥0.01. Indels were additionally removed to reduce uncertainty in true variable sites by poor alignment to the reference genome.

### 2.3 Environmental data

Biologically relevant climate variables including average rainfall, average humidity, average minimum and maximum temperature difference, average minimum temperature and average maximum temperature were obtained for each collection site from interpolated raster layers composed of values reported by Empresa de Transmisión Eléctrica Panameña (ETESA). All available data points from 2010 to 2017 representing 50–60 meteorological stations across Panama were averaged. Normalized difference vegetation indexes (NDVIs) for Panama were obtained from MODIS vegetation indices 16‐day L3 Global 250 m products (NASA, USA) with values averaged over all available images from 2010 to 2017. Human population density values were obtained from Instituto Nacional de Estadística y Censo 2010. Raster layers for generalized dissimilarity models (GDMs) and gradient forest (GF) analyses were created for each variable by inverse distance interpolation across the extent of Panama to a resolution of 0.05 pixels in QGIS version 2.18.15 (QGIS Development Team, 2019).

The collinearity and covariance of the environmental data was assessed the R Stats package (R Core Team, 2019). One variable, average minimum and maximum temperature difference, was removed from analysis because it was highly correlated with the other temperature variables (>0.8 correlation coefficient). All other variable comparisons had a correlation coefficient below 0.7 and were retained for analysis (Table S2).

### 2.4 Analysis of population structure

FastStructure was applied to all loci to infer the ancestry proportions of *K* modelled populations (Raj et al., 2014). The optimal model complexity (K*e) was chosen to be two populations using the python script chooseK.py and confirmed by a PCA of all loci performed with the R package PCAdapt (Luu et al., 2016) (see Section 2.5 below). FastStructure analysis with a logistic prior was also applied to 2630 SNPs shared with a worldwide SNP data set representing *Ae. aegypti* from 26 different countries (Gloria‐Soria et al., 2018; Kotsakiozi, Evans, et al., 2018; Kotsakiozi, Gloria‐Soria, et al., 2018; Pless et al., 2017; Saarman et al., 2017). Heterozygosity of each population was calculated in R (R Core Team, 2019) using the function in the vcfR package (Knaus & Grünwald, 2017).

### 2.5 Analysis of local environmental adaptation

To identify loci with a signal of selection differentiated across regional environmental conditions, three methods with different underlying algorithms and assumptions were applied. Two environmental association analysis (EAA) approaches, redundancy analysis (RDA) and latent factor mixed models (LFMM) were implemented to identify loci associated with environmental predictors. RDA uses multivariate regression to detect genomic variation across environmental predictors as expected from a multilocus signature of selection (Forester et al., 2018). In comparison, LFMM is a univariate approach which models background variation using latent factors, while simultaneously correlating the observed genotype frequencies of individuals to each environmental variable (Frichot et al., 2013). Before implementation of RDA, missing genotype values were imputed as the most common across all individuals. Loci which were strongly correlated with environmental predictors were then identified through multivariate linear regression of the genomic data with the environmental variables followed by constrained ordination of the fitted values as implemented with the RDA function in the R package Vegan (Oksanen et al., 2018). Multicollinearity of the data was verified to be low as indicated by genomic inflation factors ranging from 1.31 to 5.80. Candidate loci were then identified as those which contribute most to the significant axes as determined by *F* statistics (Legendre et al., 2011). To account for population structure, we applied two latent factors to our LFMM analysis based on the PCA and scree plots of proportion of explained variance produced with PCAdapt (see below). As per recommendations to improve power, we filtered our data before analysis to include only sites with a MAF >5% and analysed our data with five separate LFMM runs, each with 20,000 cycles after an initial burn‐in period of 10,000 cycles. Median *Z*‐scores were calculated from the five runs and Bonferroni corrected for multiple tests, before loci significantly correlated with environmental variables were identified based on a false discovery rate of 10% using the Benjamini–Hochberg procedure outlined in the program documentation. Visualization of the Bonferroni‐adjusted probability values for the loci correlated with each environmental factor revealed that the majority of probability values were at a flat distribution, while those correlated with environmental variables were within a peak close to 0, indicating that confounding factors were under control. In addition to the two EAAs, PCAdapt was applied to identify loci putatively under selection pressure because they deviate from the typical distribution of the test statistic *Z* (Luu et al., 2016). Two *K* populations were chosen to account for neutral population structure in the data based on scree plots of the proportion of explained variance which revealed that populations from the region of Bocas del Toro form a distinct genomic cluster (Figure 1 and Figure S1). The position and function of the candidate loci identified by all three implemented methods, RDA, LFMM and PCAdapt, were mined using the genomic resource of VectorBase (www.vectorbase.org). Average R_2_ values as a measure of linkage disequilibrium were obtained between the closely clustered candidate loci on the right arm of chromosome two (positions 2:462605993–463350786) and visualized as a heat map using the LDheatmap package in R (Shin et al., 2006). Values were plotted for comparison to the chromosome‐wide averages, obtained from a pairwise R_2_ matrix generated in PLINK v1.90 (Purcell et al., 2007) after the data were thinned by 10 kb.

### 2.6 Distribution of candidate loci across geographical space

Both putatively neutral and adaptive genomic variation were visualized across geographical space using GDM and GF analysis (Fitzpatrick & Keller, 2014). GDM is a regression‐based approach which maps allelic turnover using nonlinear functions of environmental distance in relation to *F*_ST_ genetic distance. In comparison, GF uses a machine learning regression tree approach. Through subsetting the genomic and environmental data, the algorithm determines the degree of change for each allele along an environmental gradient and calculates the resulting split importance. Allelic turnover was investigated for both a set of reference SNPs, not expected to be under selective pressure, as well as the loci putatively involved in local adaptation as jointly identified by LFMM, PCAdapt analysis and RDA. SNPs representative of neutral variation included those not identified as a candidate outlier by any of the three methods. So as to reduce the data set and avoid inclusion of strongly linked loci, SNPs were thinned by a distance of 10 kb, an appropriate cut‐off as indicated by the calculation of R_2_ linkage disequilibrium values for this data set (Figure S2).

To perform GDM analysis, the R program StAMPP (Pembleton et al., 2013) was used to generate the input *F*_ST_ matrixes and BBmisc (Bischl et al., 2017) used to rescale the distances between 0 and 1. Environmental and genetic distance data were converted to GDM format and analysis performed using the R package GDM (Manion et al., 2018). GF analysis (Ellis et al., 2012) was implemented on a matrix of minor allele frequencies for each SNP for both the reference and candidate data sets, obtained through VCFtools (Danecek et al., 2011). Both SNP data sets only included loci present in at least 11 of 14 populations to ensure robust regression. The model was fitted with 2000 regression trees, a correlation threshold of 0.5 and variable importance computed by conditional permutation with a distribution maximum of 1.37. Both analyses included Moran's eigenvector map (MEM) variables which are weightings derived from the geographical coordinates of sampling locations used to model unmeasured environmental variation and geographical distance analogous to latent factors (Fitzpatrick & Keller, 2014). To visualize the patterns in allele variation across space, PCA was used to reduce the variability into three factors. The difference in genomic composition was mapped across the landscape of Panama by assigning the three centred principle components to RGB colours; similar genomic composition across the environmental and geographical space is indicated by a similar colour shade. The difference in allele turnover for the reference and candidate data sets was characterized to explore whether allelic turnover was greater than predicted under neutral expectations. Exploration was achieved by comparing and visualizing the compositional turnover of allele frequencies for both reference and candidate SNP data sets across geographical space using a Procrustes superimposition on the PCA ordinations. Finally, so as to further compare the composition of putatively adaptive loci across the regions of Panama, a FastStructure analysis was applied as described above specifically to the candidate loci identified by all three implemented methods, RDA, LFMM and PCAdapt.

## 3 RESULTS

### 3.1 Characterization of sequence variation in *Ae. aegypti*

We processed 70 *Ae. aegypti* individuals with hybridization capture‐based enrichment from 14 localities widespread across Panama. An average number of 27,351,514 reads were mapped to the genome for each individual with 62% of these targeted to the designed capture regions. The mean coverage depth per individual was approximately 74×. After applying stringent quality filters, 371,307 SNPs were identified throughout all captured regions for downstream analyses.

### 3.2 Global and local population structure of *Ae. aegypti*

Our large SNP data set allowed us to examine population structure across both global and local scales. Comparison of global population structure was achieved by comparing a subset of 2630 of our SNPs from Panama that were shared with a previously acquired *Ae. aegypti* SNP data set from 26 other countries worldwide (Gloria‐Soria et al., 2018; Kotsakiozi, Evans, et al., 2018; Kotsakiozi, Gloria‐Soria, et al., 2018; Pless et al., 2017; Saarman et al., 2017). FastStructure analysis revealed that the number of model components and model maximum likelihood was maximized by assigning each individual to between *K* = 4–6 populations (Figure S3). Similar to that reported previously, we found that the new world variation is composed of a distinct admixture of populations including African and Asian sources at higher values of *K* (Kotsakiozi, Evans, et al., 2018; Kotsakiozi, Gloria‐Soria, et al., 2018) (Figure S3). Individuals from Panama, Costa Rica, Colombia, the Caribbean islands and populations from Arizona and Texas in south‐western United States were consistently composed of a similar composition throughout each possible value of *K* (Figure S3). Thus, *Ae. aegypti* from Panama were genetically similar to those found throughout the Americas, consistent with a strong geographical component to the distribution of genetic variation across the world (Rašić et al., 2014).

Within Panama, the much larger data set including all 371,307 SNPs highlighted significant population structure. There were two major genomic clusters (Figure 1b,c) that distinguished individuals from Bocas del Toro Province in the western Caribbean region compared to individuals from all other regions across Panama, revealed on both FastStructure analysis and PCA of all SNPs. In addition, *Ae. aegypti* from the eastern Azuero Peninsula also appeared somewhat genetically discrete (Figure 1c). All areas of Panama, including sampling locations on the Azuero Peninsula, had similar levels of heterozygosity, and therefore, the population differences we observed are not expected to result from differences in demography, that is a population bottleneck or insecticide spraying treatment, which is irregularly applied during epidemics to target adults only within the urban areas of Panama (Figure 1b).

### 3.3 Genomic evidence for local adaptation in *Ae. aegypti* in response to environmental heterogeneity across Panama

**FIGURE 2:**
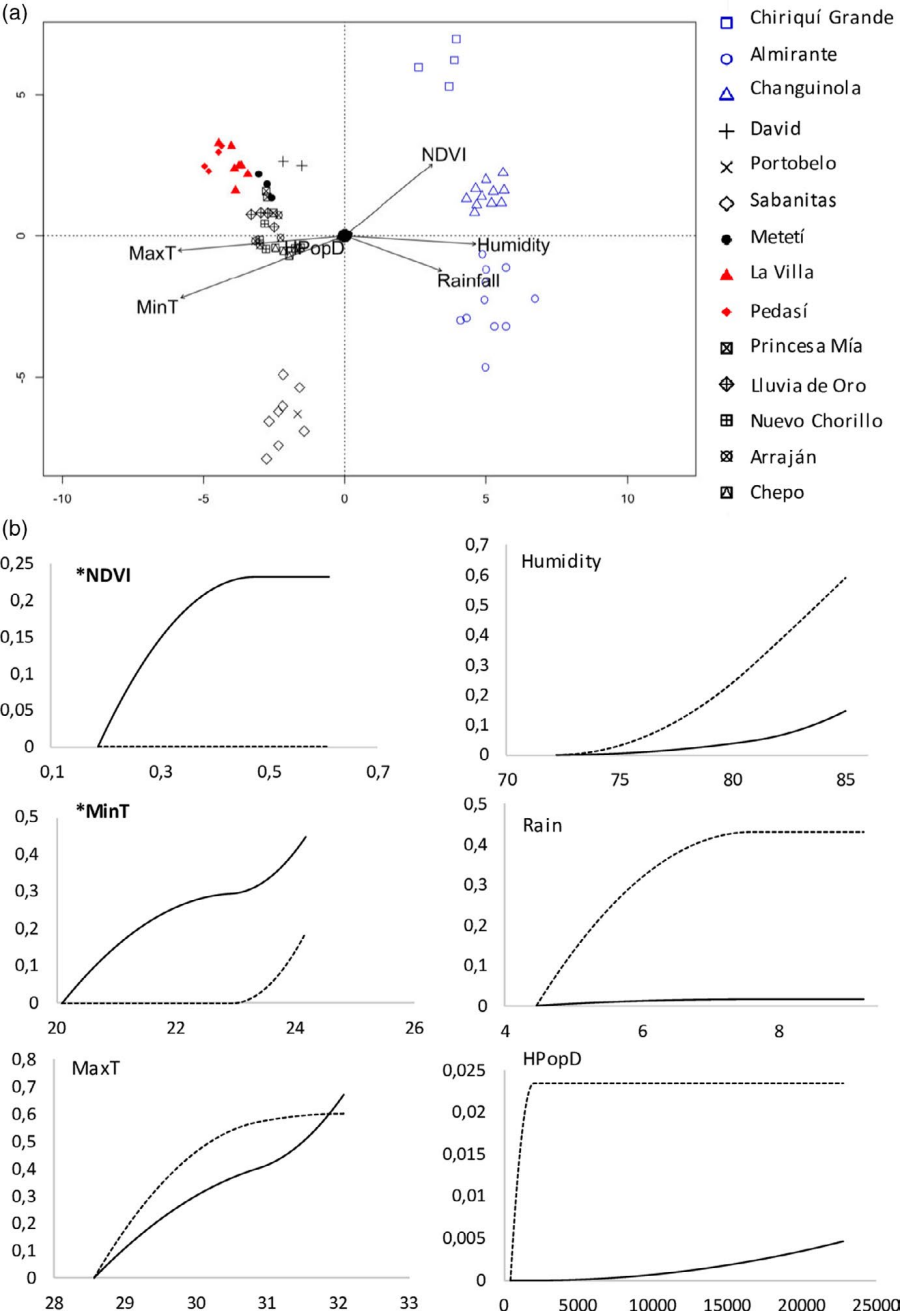
Putative adaptive variation in *Aedes aegypti* is partitioned between wet and dry tropical environments and associated with temperature and vegetation indices: (a) Ordination triplot of the first two constrained ordination axes of the redundancy analysis representing SNPs either positively or negatively associated with the environmental variables as depicted by the position of the arrows. *Ae. aegypti* from the wettest region (blue) and driest region (red) are highlighted. (b) Compositional turnover splines for GDM analysis for the reference loci that are putatively neutral (dashed line) and the 128 candidate loci with a signal of local adaptation (black line) in association with NDVI, average minimum temperature (MinT), average maximum temperature (MaxT), average humidity (Humidity), average rainfall (Rain) and human population density (HPopD). For each plot, the y‐axis represents the compositional turnover value and the x‐axis represents the environmental data. A change in allele frequency relative to the reference loci is seen in the putatively adaptive alleles with increasing values of NDVI and MinT, marked in bold with an asterisk

If locally adapted, we would expect populations of *Ae. aegypti* to harbour genomic loci with a signal of selection that are correlated with the local environmental conditions, after taking the underlying population structure into account. As a first step, we applied RDA to jointly identify candidate outlier loci and to assess how candidate variation was partitioned among the different environmental variables. In this analysis, we tested a number of environmental variables including NDVI, average rainfall, average humidity, average minimum and maximum temperature, and human population density. RDA identified 1154 candidate SNPs with a genomic signal of local adaptation, which we used to visualize putatively adaptive variation on ordination plots. Overall, there was a partitioning of alleles associated with dry tropical and wet tropical conditions. For example, the position of sampled individuals on the RDA ordination plots, in relation to the depicted environmental variables, revealed that the candidate genotypes of *Ae. aegypti* from the wet tropical regions of Almirante and Changuinola in Bocas del Toro Province were positively associated with average humidity and average rainfall. Those from the wet tropical region of Chiriquí Grande in Bocas del Toro were also positively associated with increasing NDVI and negatively associated with higher temperatures (Figure 2a). In comparison, the candidate genotypes of individuals from dry tropical regions of Panamá Province (i.e. Princesa Mía, Lluvia de Oro, Nuevo Chorrillo), Los Santos (i.e. La Villa de Los Santos, Pedasí), Darién (i.e. Metetí) and David in Chiriquí Province were somewhat positively influenced by both temperature variables and negatively associated with wet and vegetated conditions. Putatively adaptive variation in individuals from Sabanitas and Portobelo in the province of Colón, which receive high rainfall but higher temperatures and lower vegetation cover than in Bocas del Toro Province, was associated with intermediate temperature and vegetation conditions.

RDA is robust in detecting adaptive processes that result from weak, multilocus effects across a range of demographic scenarios and sampling designs (Forester et al., 2018). However, a proportion of the 1154 candidate loci identified through this single analysis were likely false positives. Thus, rather than reflecting local adaptation, the strongly skewed frequency differences could be reflective of demographic processes such as hierarchical population structure, isolation by distance, allele surfing on range expansion and background selection, or the coincidental associations of allele frequencies to environmental variation or even covariance to other environmental factors not included in the analysis (Rellstab et al., 2015). To further refine our identification of putatively adaptive loci, we identified candidates using two additional methods, PCAdapt and LFMM. Both are considered less sensitive to confounding demography due to their ability to account for population structure or unobserved spatial autocorrelation in the data (de Villemereuil et al., 2014). The three methods identified different numbers of putatively adaptive loci. Compared to the 1154 outlier SNPs identified by RDA, PCAdapt identified 352 SNPs (Figure S4), whereas LFMM analysis identified 3426 outlier SNPs with a signature of selection widespread across the genome and associated with the environment, respectively (Figure S5).

**FIGURE 3:**
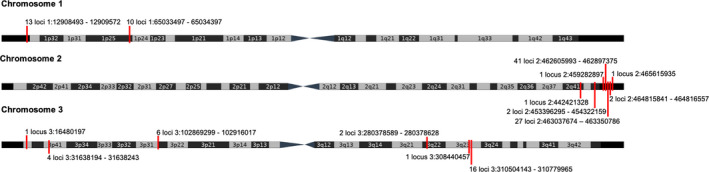
The candidate loci form 15 distinct clusters widespread across the *Aedes aegypti* genome. The location of the 128 candidate loci within each of the three chromosomes of the *Ae. aegypti* genome that were identified as putatively involved in local adaptation, shared across selection and EAA. At each location, the number of loci is identified and their genomic coordinates provided

Across all three methods, there were 128 SNPs consistently identified as outliers with a signal of selection and correlated with environmental variables, providing greater confidence that these loci are located in or close to genomic regions possibly involved in local adaptation. These candidate SNPs fell into 15 distinct clusters, suggesting that linkage disequilibrium was driving some of the observed patterns (Figure 3). In particular, 67 of the candidate SNPs composing seven genes are closely clustered within a ~0.77 Mb region at the end of the right arm of chromosome two spanning positions 2:462605993–463350786. Patterns of linkage disequilibrium across chromosome two are complex, but the region with the candidate loci had higher average levels of R_2_ than the background average (Figures S6 and S7). Overall, the average R_2_ value across chromosome two was 0.017 in comparison to an average R_2_ value of 0.113 across the candidate positions of interest. Since population history is controlled for in our identification of candidates, a pattern of linkage disequilibrium at the genomic location 2:462605993–463350786 is likely to represent either natural selection or a potential genomic rearrangement associated with the environment. Overall, the 128 SNPs fell into 17 genes, 11 of which are annotated as involved in structural functions, enzyme activity and metabolism (Table S3). None of these genes are known to be involved in the development of insecticide resistance in populations of *Aedes* mosquitoes.

We further narrowed down which of the environmental variables contributed most to the partitioning of genomic variation using a combination of GDM and GF analyses. Both approaches allowed us to visualize the allelic turnover of these putatively adaptive loci in relation to each environmental variable. The environmental variables that contributed the greatest variance to both GDM and GF model on analysis of the 128 candidate loci were minimum and maximum temperature (Figure S8 and Table S4). GDM analysis revealed that an increase in average minimum temperature accompanied a large change in putatively adaptive allele frequencies, visualized as a smooth curve accumulating in a steeper incline at the higher temperature range (Figure 2b). In comparison, GF turnover plots show a steeper incline at the mid‐range for both average minimum and maximum temperature (Figures S9). GDM analysis also revealed a distinct frequency change in putatively adaptive alleles with increasing NDVI, although the change in allele frequency was relatively minor compared to that of minimum temperature (Figure 2b). In comparison, a low to negligible difference in allele frequency was observed in association with average rainfall, average humidity and human population density. Therefore, the variation in putatively adaptive allele frequencies between populations from dry tropical and wet tropical environments of Panama appears largely driven by differences in temperature and NDVI.

### 3.4 The geographical distribution of loci associated with the environment

**FIGURE 4:**
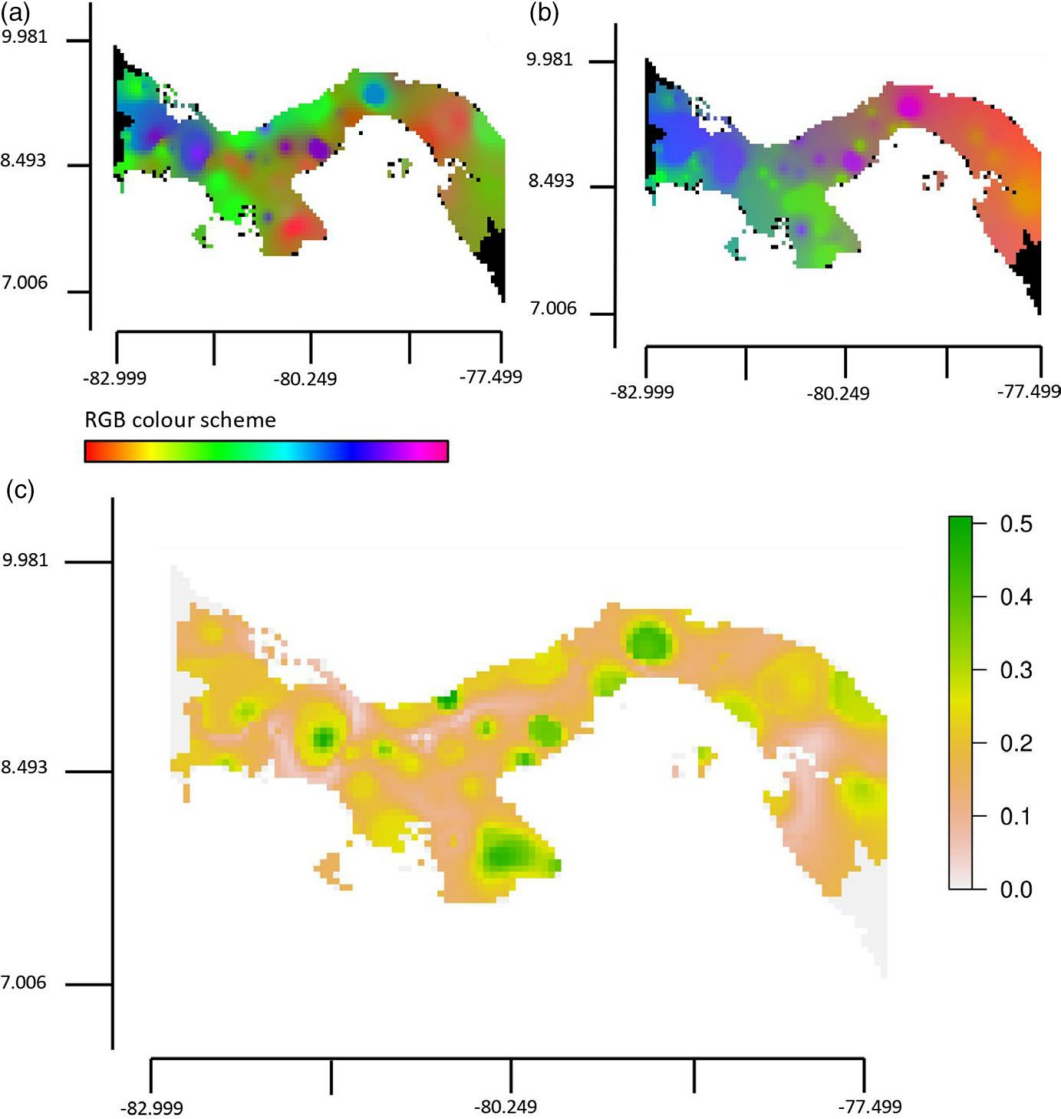
Patches of local adaptation are revealed on comparison of putative neutral and adaptive variation across geographical space. RGB maps of compositional allele frequency turn over across geographical space based on GDM analysis of (a) putatively neutral loci, (b) the 128 candidate loci with a signal of local adaptation and (c) the difference in allele compositional turnover between the putatively neutral reference loci and putatively adaptive candidate data set using a Procrustes superimposition on the PCA ordinations. On maps (a) and (b), the dissimilarity between allele composition is depicted by an increasing divergent colour spectrum. Locations with a similar allele composition are a similar colour based on the RGB colour scheme. On map (c), the scale represents the distance between the allele compositional turnover of the reference and candidate SNP data sets, with higher distances indicating areas that are potentially experiencing local adaptation

Across our 128 candidate SNPs, we used GDM and GF analysis to visualize the change in frequencies across Panama and therefore the geographical landscape features which increase or decrease the genomic signature of local adaptation in relation to the environment. GDM analysis presented a smoother turnover in the geographical distribution of putatively adaptive loci than that of putatively neutral loci as indicated by a smoother transition in the colour palette between proximal geographical locations (Figure 4a,b). For example, there was similarity in the colouring and therefore allele composition between wet tropical regions along the Caribbean coast (i.e. the mainland/islands of Bocas del Toro, Chiriquí, and both the inland and Caribbean coastal regions stretching from Bocas del Toro through Veraguas to Colón). Similarly, there was greater continuity between dry tropical areas including David in Chiriquí, the eastern Azuero Peninsula (i.e. La Villa de Los Santos and Pedasí), the Pacific coastal regions stretching from the Azuero Peninsula through Coclé to Panamá, and the Darién (i.e. Metetí), indicating that these environments share putatively adaptive alleles. Patterns in the data were less distinct for GF analysis but the geographical distribution of putatively adaptive variation agreed with the GDM analysis in that there was a continuity in the allele composition between the eastern Azuero Peninsula and dry tropical Pacific coastal regions, distinct from the wet tropical regions along the Caribbean coast (Figure S10).

Allele frequency turnover as predicted under neutral conditions and a scenario of local adaptation involving the candidate loci were compared across geographical space to identify locations that show the greatest disparity. These reflected the populations within Panama expected to be experiencing a strong genomic signal of local adaptation. Their comparison revealed multiple patches of potential local adaptation widespread across Panama, with a palpable patch occurring in the Azuero Peninsula, as indicated by a high distance between the patterns of predicted compositional allele frequency turnover (Figure 4c). A genomic signal of local adaptation was not identified in the region of Bocas del Toro. Since this region has a unique genomic variation supportive of strong population structure within Panama and also a distinct climatic regime, it is likely that the co‐correlation of neutral and adaptive environmental variation across our sampling design hindered the visualization of possible patches of local adaptation in Bocas del Toro. This conclusion was supported by FastStructure analysis of the 128 putatively adaptive loci, which revealed that *Ae. aegypti* from the wet tropical region Bocas del Toro has a distinct allele composition composed of alleles assigned to a distinct composition of *K* populations, including unique alleles in addition to those shared broadly across the dry tropical regions of Panama (Figure S11). Although the Talamanca mountain range was documented as a natural geographical barrier to dispersal across the region of Bocas del Toro for some *Anopheles* mosquitoes (Loaiza & Miller, 2019), this was not expected to hinder gene flow in *Ae. aegypti*, since human‐assisted movement of this mosquito occurs via the local transport network (Bennett et al., 2019). Partitioning of the genomic data into *K* = 6 populations revealed that Sabanitas on the Caribbean coast, which is subject to intermediate climate conditions, shared some of the distinct alleles present in Bocas del Toro. Moreover, individuals from the eastern Azuero Peninsula, the driest and least vegetated region of Panama, were also somewhat distinct from other sampled regions since they had reduced levels of admixture.

## 4 DISCUSSION

This is one of the first studies to investigate the fine‐scale genomic architecture of *Ae. aegypti* across a heterogenous tropical landscape. On a regional scale, Panamanian populations of *Ae. aegypti* are genetically similar to other Central and Caribbean American populations highlighting high dispersal potential and recent gene flow in this invasive species; however, this similarity belies a more complex local genomic architecture. Across Panama, genomic variation was not structured randomly, with the isolated Bocas del Toro region showing significant overall population differentiation. Across the rest of Panama, populations are more homogeneous suggesting higher levels of gene flow, likely facilitated by the dispersal of *Aedes* mosquitoes in used tyres that are traded along the Pan‐American highway, which is the only major transport route across the country connecting the different regions and all major towns (Bennett et al., 2019). Nonetheless, a subset of genomic variation was differentially distributed with evidence of localized adaptation across a relatively small number of SNPs and over a relatively fine geographical scale. Genomic variation in these SNPs was strongly correlated with temperature and NDVI. Both these abiotic variables were previously identified as important in predicting large‐scale *Aedes* distribution patterns (Kraemer et al., 2015). Temperature is important for egg laying, development and survival of *Ae. aegypti* in larval habitats (Brady et al., 2014) and likely to promote selection to thermal tolerance at the adult stage to resist diurnal and inter‐seasonal variation (Brady et al., 2013). Vegetation is considered an important variable that contributes to oviposition cues (Afify & Galizia, 2015), feeding dynamics (Tun‐Lin et al., 2000) and microhabitat characteristics such as local moisture supply and shade (Lounibos et al., 2010; Vezzani et al., 2005). Although correlational, the genomic patterns raise an important question: Are *Ae. aegypti* mosquitoes differentially adapted to local environments?

The possibility of climatically adapted populations of *Ae. aegypti* is not without precedence. Data on a wide range of organisms with varying dispersal abilities (Ahrens et al., 2019; Exposito‐Alonso et al., 2018; Geue et al., 2016; Hancock et al., 2011; Miller et al., 2019; Oliveira et al., 2018; Zhen et al., 2017) demonstrate that even well‐connected populations can adapt to environmental differences and habitat heterogeneity across narrow spatial scales. Similar to other landscape genomics studies on plants (Pais et al., 2017; Prunier et al., 2011; Shryock et al., 2015), insects (Dudaniec et al., 2018) and vertebrates (Bay et al., 2018), we have found a signal of local environmental adaptation across a small number of loci. The inability to identify more putative regions under selection may be the result of the analytical difficulties in distinguishing weak multilocus signatures from the genomic differentiation introduced by genetic drift and demography (Forester et al., 2018; Hoban et al., 2016). However, selection on just a few loci with large effects is expected when migration is high since large effect loci are better able to resist the homogenizing effects of gene flow (Savolainen et al., 2013). These few regions are expected to have a strong impact on fitness in one environment over the other because the allele with the highest fitness is expected to spread to all populations if this condition is not met (Savolainen et al., 2013). Our findings provide clear, testable hypotheses moving forward. For example, if the genomic regions we identified are adaptive, then we expect genotype specific survival under different environmental conditions, which can be tested in a common garden with reciprocal transplant experiment.

The presence of locally adapted populations of *Ae. aegypti* could have a significant impact on the future arboviral disease landscape. Climate variables, most notably precipitation and temperature associated with altitudinal and latitudinal clines, are able to drive population differentiation in both *Anopheles* mosquitoes and *Drosophila* flies (Cheng et al., 2012; Kapun et al., 2016; Love et al., 2016; Simard et al., 2009). In the former, the Anopheles gambiae species complex is hypothesized to have radiated through ecological speciation driven by adaptation to aridity and in response to larval habitat competition. This has led to a series of ecotypes with semi‐permeable species boundaries (Ayala et al., 2014). Interestingly, adaptation to the climate in both *Drosophila* and *Anopheles* is facilitated by the presence of chromosomal inversions, which contribute to local adaptation by reducing recombination and allowing the co‐adaptation of linked genes (Redmond et al., 2020). Although we lack the data resolution to investigate further, up to 52.3% of our candidate loci cluster at the end of chromosome arm two, raising the possibility there is a genomic rearrangement such as an inversion, translocation or fusion/fission linking these loci. The large and repetitive genome of *Ae. aegypti* complicates the accurate identification of inversions, but 23 microinversions have been recently described using long read‐sequencing (Redmond et al., 2020), including the inversion 2qam (2:456306790–456341898), which is located close to where we see a clustering of our candidate loci, although none of the candidates fall within this specific region of chromosome two. Whether these candidate loci both confer an environmental adaptation and fall within a genomic rearrangement is a subject for future investigation.

Inversions in *Anopheles* have been directly linked to traits such as thermal tolerance and oviposition site preferences that can impact on disease transmission dynamics (Fouet et al., 2012; Sanford et al., 2013). In particular, the resulting differences among *Anopheles* ecotypes in anthropophily and adult resting behaviour have a significant impact on malaria transmission risk (White et al., 2011). Thus, at the most basic level, differentially adapted population variants of *Ae. aegypti* across Panama could have different abilities to vector arboviral disease (Chouin‐Carneiro et al., 2016; Gonçalves et al., 2014; Hugo et al., 2019; Lounibos & Kramer, 2016; Roundy et al., 2017; Vega‐Rúa et al., 2014).

The presence of locally adaptive *Ae. aegypti* populations could also have a profound impact on how populations respond to the recent introduction of the Asian tiger mosquito, *Ae. albopictus*. *Ae. albopictus* is an ecologically similar mosquito that is undergoing a global range expansion further complicating the arbovirus disease landscape (Lounibos & Kramer, 2016). For example, in the United States and Bermuda, *Ae. albopictus* has displaced resident *Ae. aegypti* in some areas but these two are able to persist together in some environments (Bargielowski et al., 2016; Kaplan et al., 2010). *Ae. albopictus* colonized Panama about 10 years ago and has spread rapidly across the country (Miller & Loaiza, 2015). Given that these mosquitoes have different abilities to vector disease and are competent for a different range of viral pathogens, changes in their distributions are likely to influence the arboviral disease landscape (Pereira Dos Santos et al., 2018). The factors that facilitate co‐occurrence are still unclear (Lounibos & Juliano, 2018), but the local adaptation of the resident *Ae. aegypti* populations should be considered in the competitive outcome, given that locally adaptive populations may better persist in the presence of an invading competitor (Pinsky, 2019).

Environmental adaptation needs to be considered in spatially predictive models. Currently, species geographical distribution or disease prediction models incorporate a set of environmental parameters coupled with a predicted outcome on mosquito biology and abundance without considering adaptive response (Bartlow et al., 2019; Kalluri et al., 2007). Assuming that the whole population will respond to environmental precursors as a homogenous unit is erroneous when local adaptation is present and considering adaptability as a parameter, in combination with the environmental response, will improve the accuracy of future projections (Kearney et al., 2009; Kraemer et al., 2015). Furthermore, the presence of locally adapted populations threatens the efficiency of gene drive systems aimed at promoting disease resistance within mosquito populations. This is because environmental differences between sites, as well as physical geographical barriers, will restrict mosquito dispersal and therefore limit the spread of beneficial alleles or inherited bacteria (Jiggins, 2017). However, if locally adaptive alleles are well characterized, this knowledge could also potentially be exploited. A more tailored methodology could improve the gene drive efficiency of either approach since locally adapted individuals are theoretically more likely to survive to pass on the intended benefit to the next generation.

## 5 CONCLUSION

The identification of a small number of putatively adaptive genomic intervals provides exceptional experimental opportunities to determine (1) if these regions are in fact under selection due to local environmental adaptation and (2) how selection might be acting to increase the fitness and acquisition of new traits in *Ae. aegypti*, if our hypothesis is true. Defining species fitness in association with our candidate loci will allow us to untangle the interplay between genomic process, the environment and how these resolve the spatial distribution and abundance of medically important *Ae. aegypti*. Advances will be used to improve the accuracy of disease prediction models and characterize the genomic basis of adaptations with the capacity to alter the epidemiological landscape.

## CONFLICTS OF INTEREST

The authors received funding from The Edward M. and Jeanne C. Kashian Family Foundation Inc., and Nicholas Logothetis of Chartwell Consulting. There are no patents, products in development or marketed products associated with this research to declare.

## Supporting information

Fig S1‐11

Table S1

Table S2

Table S3

Table S4

Dataset S1

## Data Availability

SNP data are available in the Sequence Read Archive data repository under BioProject PRJNA639740.
