# Supplementary material for: The genomic signal of local environmental adaptation in Aedes aegypti mosquitoes
Source: Evol Appl. 2021 Feb 26;14(5):1301–13. doi: 10.1111/eva.13199 (PMC8127705; doi:10.1111/eva.13199)
Supplement: Supplementary file 1 — Fig S1‐11 [file EVA-14-1301-s002.docx]

**The genomic signal of local environmental adaptation in *Aedes aegypti* mosquitoes**

**Supplementary Material**

**Supplementary Figures**


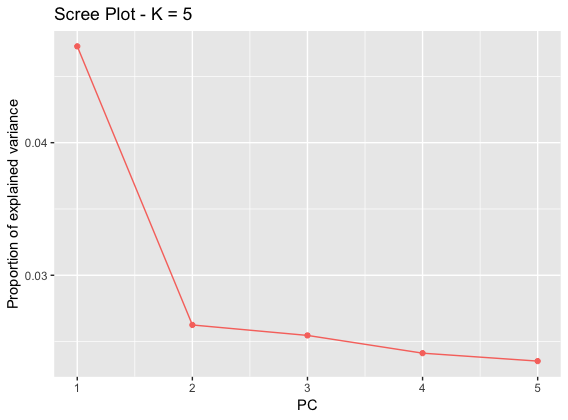


**Figure S1.** Scree plot produced in PCAdapt showing the selection of K=2 populations explains the variance in the genomic data of *Ae. aegypti*.


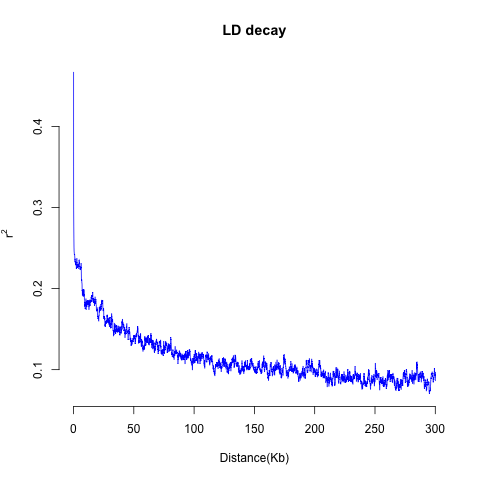


**Figure S2.** LD decay plot of R_2_ linkage disequilibrium across all loci the SNP dataset.


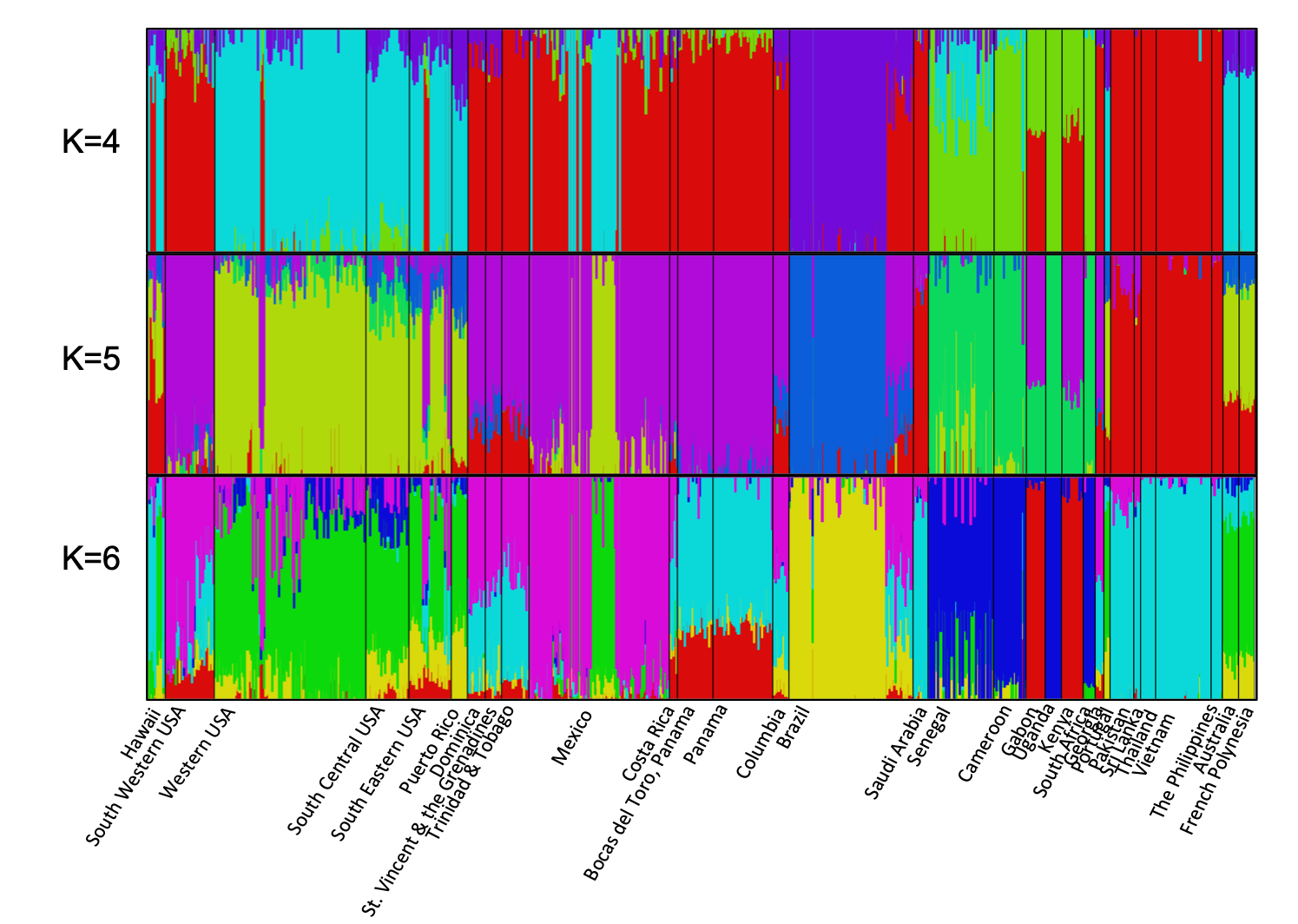


**Figure S3.** FastStructure plots comparing 2,630 SNP’s from individuals of *Ae. aegypti* from Panama to 26 other worldwide locations. Plots are provided for between K=4 populations, the best number of model components used to explain structure in data, and K=6 populations, reflecting the model complexity that maximizes the marginal likelihood. FastStructure assigns each individual to one or more K populations, as indicated by its colour. Genetically similar populations share the same colour or similar admixture composition on comparison within each separate plot.


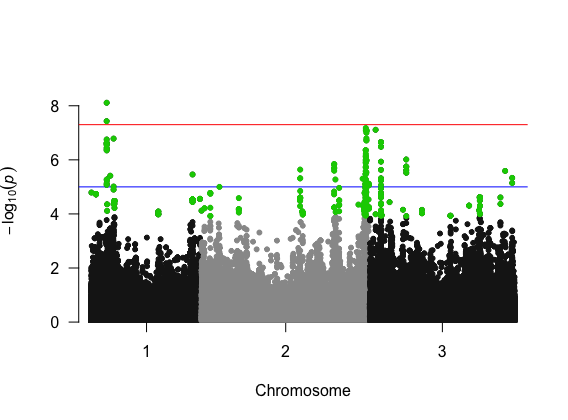


**Figure S4.** Manhattan plot showing the location of putative adaptive loci across each of the three *Ae. aegypti* chromosomes as identified by PCAdapt. Loci above the red line, which is the threshold for genome-wide significance, show highly significant signals of selection, while those above the blue line are suggestive of selection. Those identified by the analysis as candidate loci with a signal of local adaptation are highlighted in green.

**
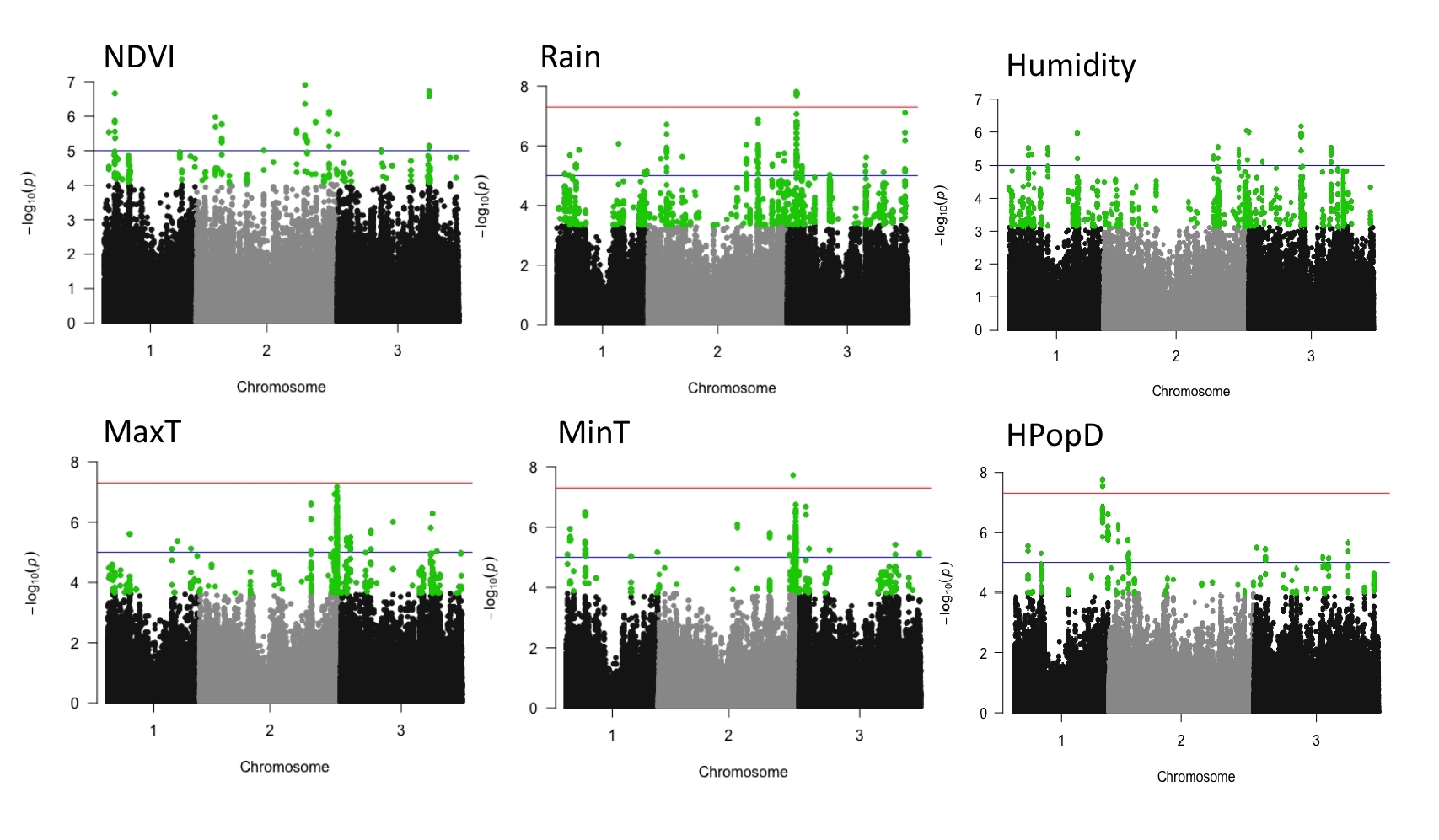
**

**Figure S5.** Manhattan plots showing the distribution of genomic loci correlated to NDVI vegetation index (NDVI), average rainfall (Rain), average humidity, average minimum rainfall (MinT), average maximum temperature (MaxT) and human population density (HPopD) across the three chromosomes of the *Ae. aegypti* genome. Loci above the red line, which is the threshold for genome-wide significance, show highly significant signals of selection, while those above the blue line are suggestive of selection. Those identified by the analysis as candidate loci with a signal of local adaptation based on a false discovery rate of 10 % are highlighted in green.


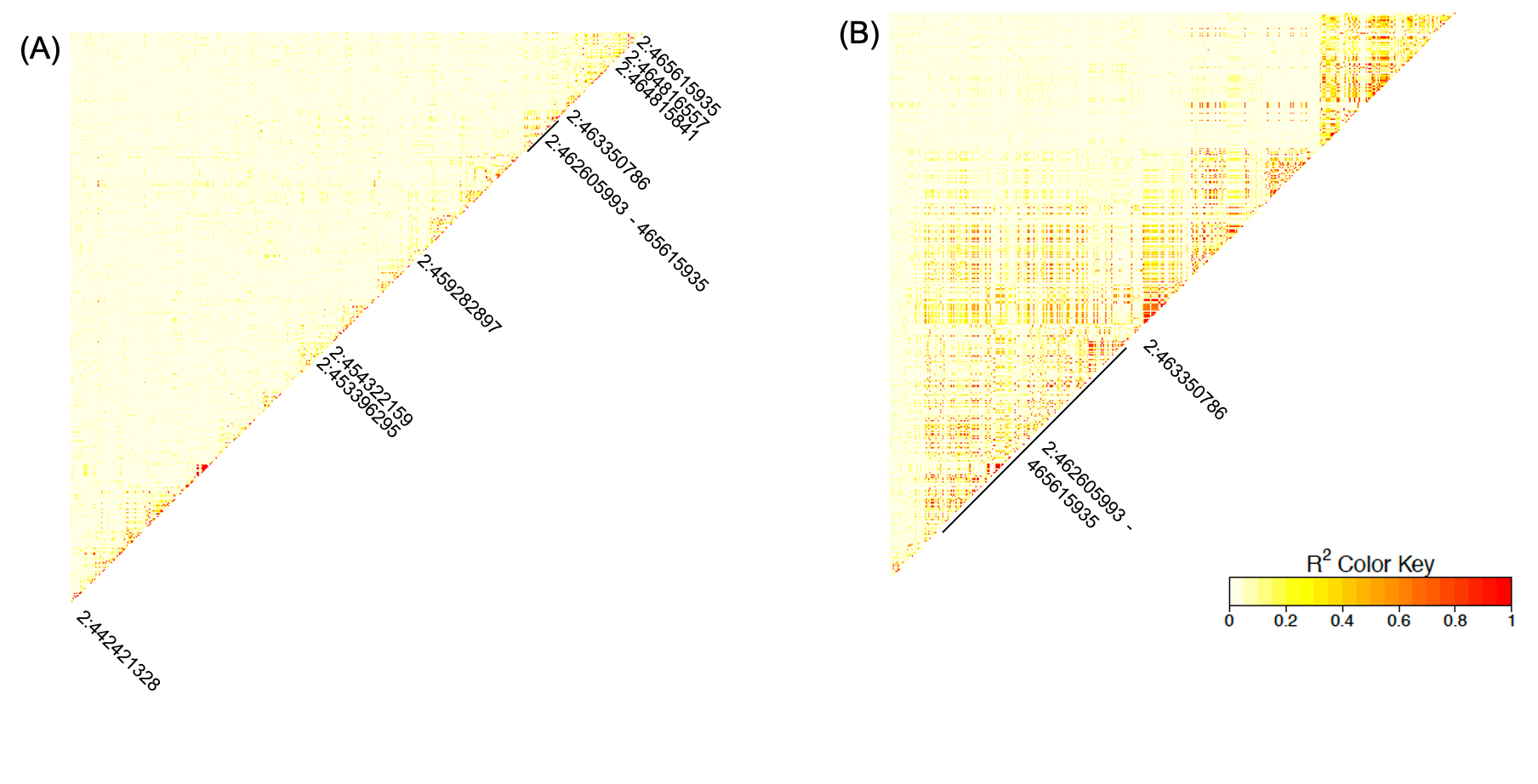


**Figure S6.** Linkage disequilibrium heat map based on a pairwise matrix of R_2_ values (A) across a region spanning 75 candidate loci that were identified on chromosome two (2:442421328 to 2:465615935) and (B) for 67 of these loci that form a dense cluster. The candidate loci in the region are indicated on each heat map.


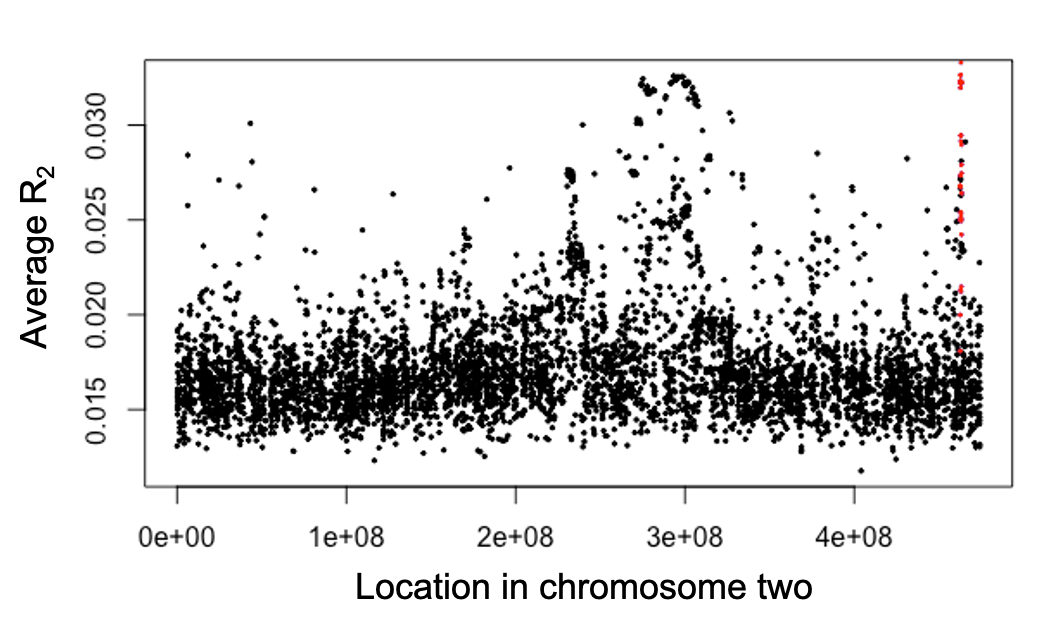


**Figure S7.** Average R_2_ across the genomic loci of chromosome two (black) in comparison to the average R_2_ between the candidate loci at the chromosome two positions 2:462605993-463350786 (red).


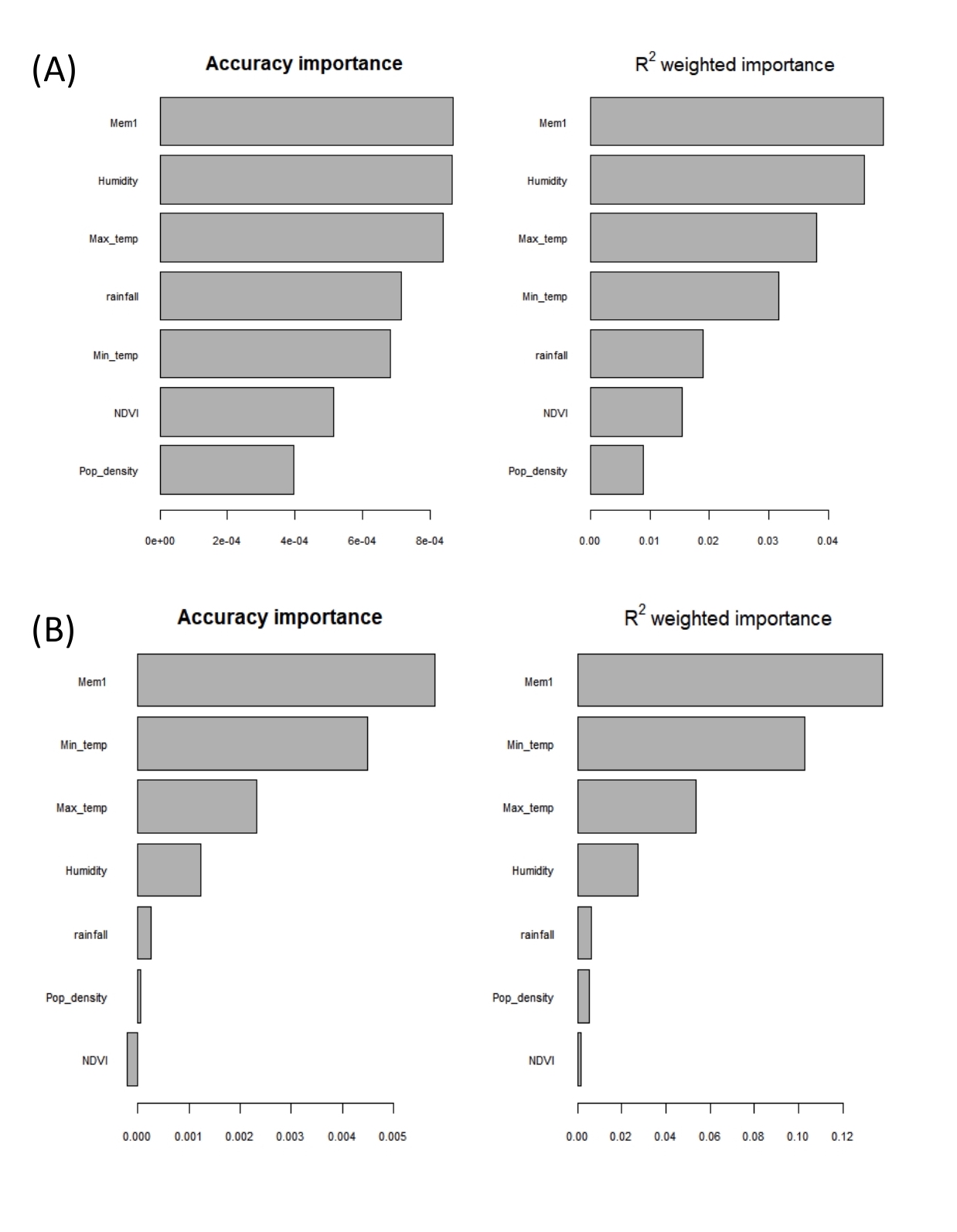


**Figure S8.** Variable importance of the environmental variables in the GF model for a. reference and b. candidate loci. Moran’s eigenvector map variables (Mem1) represent spatial effects and are the eigenvectors of a spatial weighting matrix derived from the geographic coordinates of sampling locations.


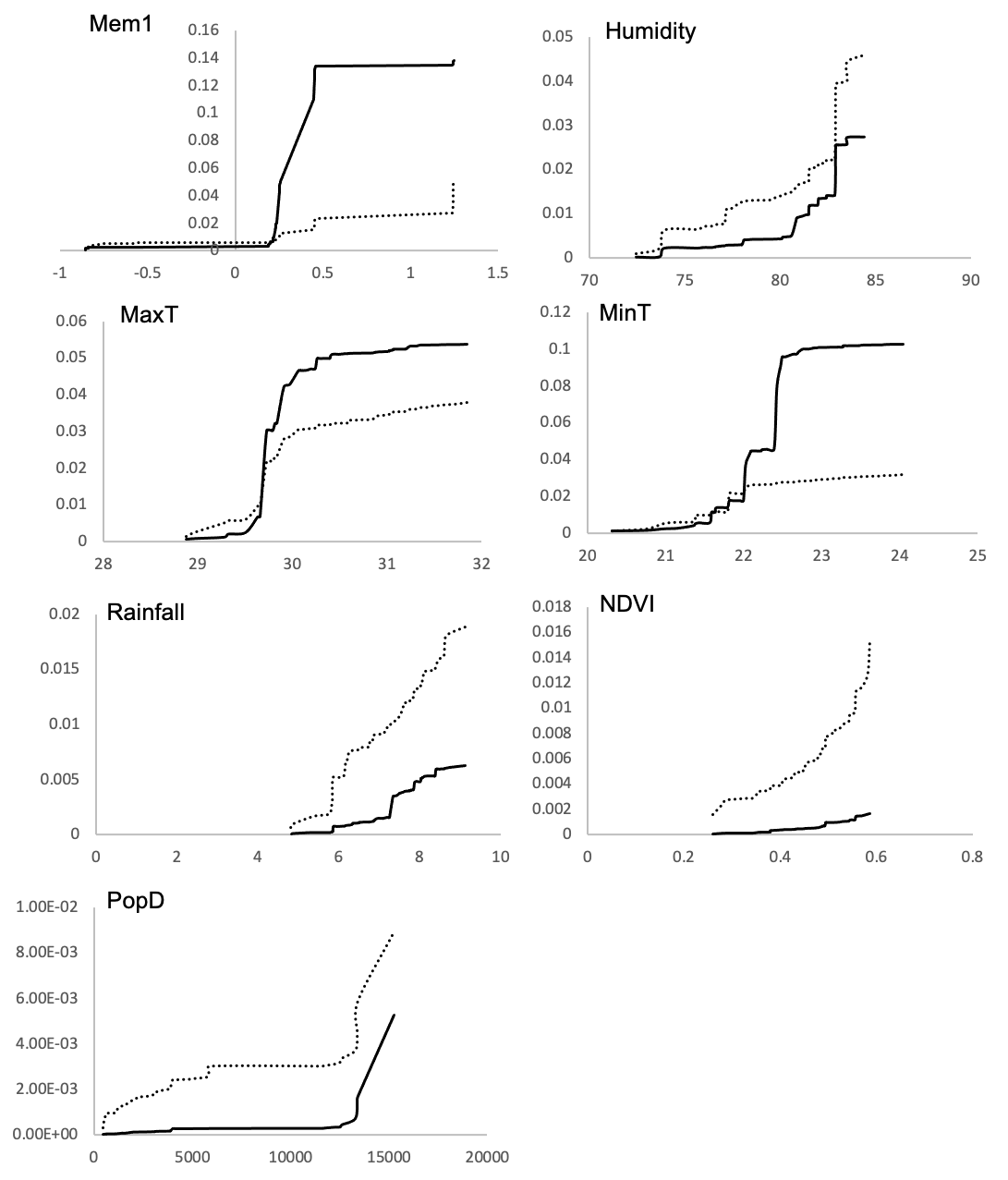


**Figure S9.** Aggregate compositional turnover plots for GF analysis depicting allele change of the reference loci that are putatively neutral (dashed line) and the 128 candidate loci with a signal of local adaptation (black line) across each environmental gradient including Moran’s eigenvector map variables (Mem1), average humidity, average maximum temperature (MaxT), average minimum temperature (MinT), average rainfall, normalised difference vegetation index (NDVI) and human population density (PopD). Moran’s eigenvector map variables (Mem1) represent spatial effects and are the eigenvectors of a spatial weighting matrix derived from the geographic coordinates of sampling locations. The maximum height of the line gives the overall change in allele frequency and therefore the relative variable importance.


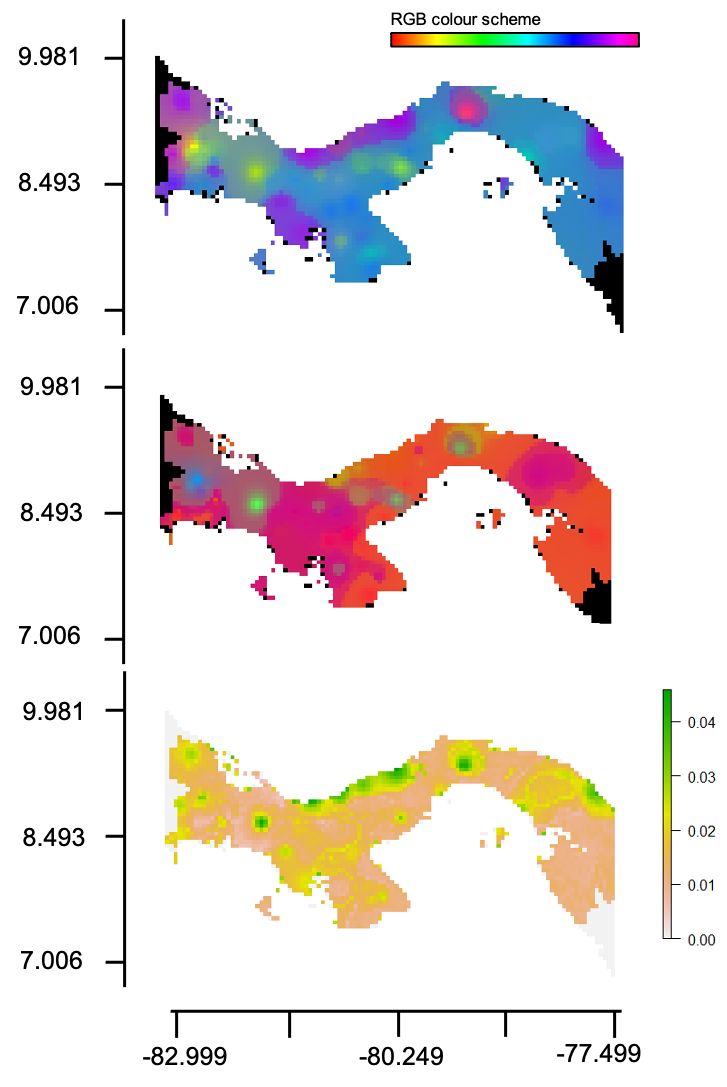


**Figure S10.** RGB maps of compositional allele frequency turn over across geographical space based on GF analysis for reference loci (above), candidate loci (centre) and the difference in allele compositional turnover between the reference and candidate dataset using a Procrustes superimposition on the PCA ordinations (below). In the above and centre map, dissimilarity between allele composition is depicted by an increasing divergent colour spectrum. Locations with a similar allele composition are a similar colour based on the RGB colour scheme. On the below map, the scale represents the distance between the allele composition of the reference and candidate SNP datasets, with higher distances indicating areas that are potentially experiencing local adaptation.


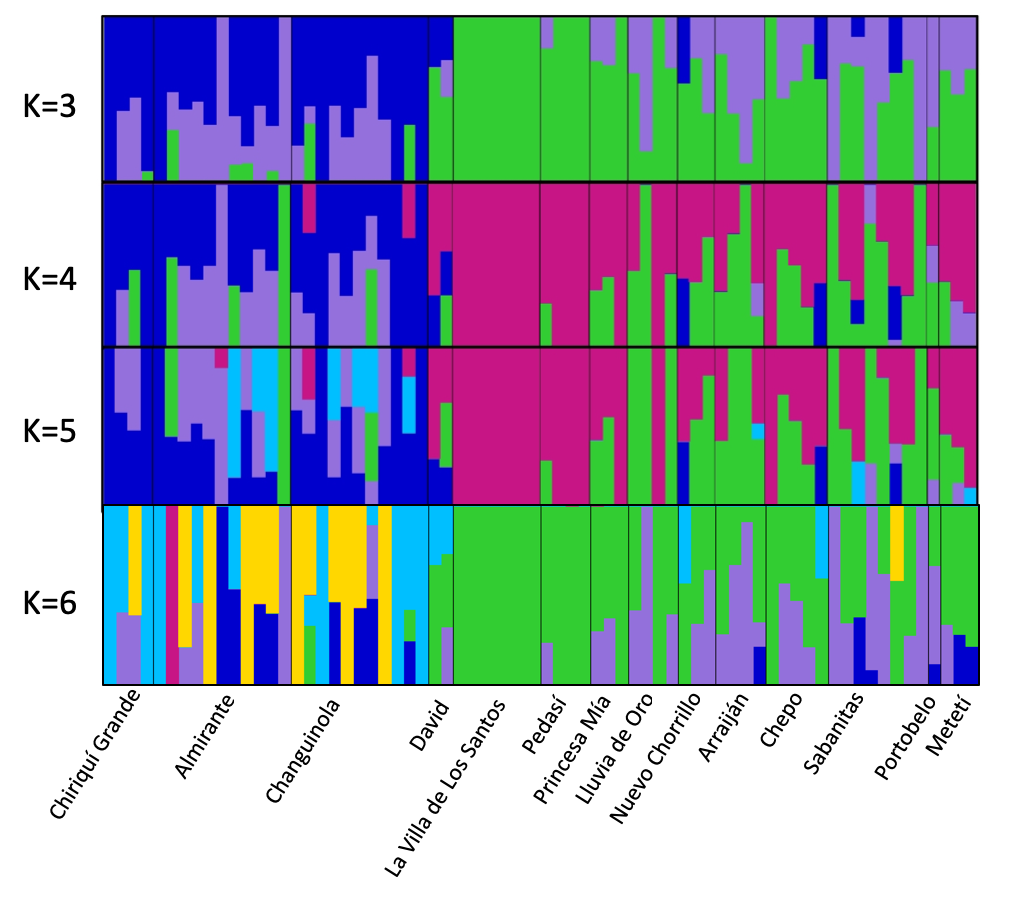


**Figure S11.** FastStructure plot of 128 candidate loci with a signal of local adaptation for between K=3 (the number of model components used to explain structure in data) and K=6 populations (the model complexity that maximizes the marginal likelihood). FastStructure assigns each individual to one or more K populations, as indicated by its colour. Genetically similar populations share the same colour or similar admixture composition on comparison within each separate plot.
